# Identification of Factors That Motivate People With Multiple Sclerosis to Participate in Digital Data Collection in Research: Sequential Mixed Methods Study

**DOI:** 10.2196/13295

**Published:** 2019-10-09

**Authors:** Astrid Karnoe, Lars Kayser, Lasse Skovgaard

**Affiliations:** 1 Department of Public Health University of Copenhagen Copenhagen Denmark; 2 Danish Multiple Sclerosis Patient Society Valby Denmark

**Keywords:** health literacy, computer literacy, mobile apps, patient participation, research design, multiple sclerosis

## Abstract

**Background:**

Digital data collection has the potential to reduce participant burden in research projects that require extensive registrations from participants. To achieve this, a digital data collection tool needs to address potential barriers and motivations for participation.

**Objective:**

This study aimed to identify factors that may affect motivation for participation and adoption of a digital data collection tool in a research project on nutrition and multiple sclerosis (MS).

**Methods:**

The study was designed as a sequential mixed methods study with 3 phases. In phase 1, 15 semistructured interviews were conducted in a Danish population of individuals with MS. Interview guide frameworks were based on dimensions from the electronic health literacy framework and the Health Education Impact Questionnaire. Data from phase 1 were analyzed in a content analysis, and findings were used to inform the survey design in phase 2 that validates the results from the content analysis in a larger population. The survey consisted of 14 items, and it was sent to 1000 individuals with MS (response rate 42.5%). In phase 3, participants in 3 focus group interviews discussed how findings from phases 1 and 2 might affect motivation for participation and adoption of the digital tool.

**Results:**

The following 3 categories related to barriers and incentives for participation were identified in the content analysis of the 15 individual interviews: (1) life with MS, (2) use of technology, and (3) participation and incentives. Phase 1 findings were tested in phase 2’s survey in a larger population (n=1000). The majority of participants were comfortable using smartphone technologies and participated actively on social media platforms. MS symptoms did cause limitations in the use of Web pages and apps when the given pages had screen clutter, too many colors, or too small buttons. Life with MS meant that most participants had to ration their energy levels. Support from family and friends was important to participants, but support could also come in the form of physical aids (walking aids and similar) and digital aids (reminders, calendar functions, and medication management). Factors that could discourage participation were particularly related to the time it would take every day. The biggest motivations for participation were to contribute to research in MS, to learn more about one’s own MS and what affects it, and to be able to exchange experiences with other people with MS.

**Conclusions:**

MS causes limitations that put demands on tools developed for digital data collection. A digital data collection tool can increase chances of high adoption rates, but it needs to be supplemented with a clear and simple project design and continuous communication with participants. Motivational factors should be considered in both study design and the development of a digital data collection tool for research.

## Introduction

### Multiple Sclerosis, Diet, and Lifestyle

People with multiple sclerosis (MS), an autoimmune inflammatory disease in the central nervous system, experience individual and complex symptom patterns (eg, fatigue, cognitive impairment, walking difficulties, pain, bowel dysfunction, and bladder dysfunction) [1-3]. Symptom severity and fluctuations affect the perception of health and quality of life, and some people with MS identify diet and other lifestyle factors as triggers for daily symptom worsening [4-6]. A possible approach to investigate correlations among MS symptoms, diet, and lifestyle would be to collect daily patient-reported data on relevant variables. However, this kind of data collection relies heavily on daily manual registrations made by participants with MS.

### Digital Data Collection

A digital, smartphone-based data collection has the potential to improve the participants’ experience and, at the same time, reduce recall bias compared with a traditional pen and paper study design [7,8]. Furthermore, previous studies indicate that the use of digital and internet-based services is high in MS populations and, in some countries (eg, the United States), higher than the internet use in a general population sample [9-11].

However, heavy manual data registration place demands on the participants, and furthermore, studies testing digital patient portals, remote care services, and symptom management solutions all find that physical MS symptoms including vision impairment can cause barriers related to adoption if not addressed in the design and development stages [9,10,12]. Although studies investigating electronic health (eHealth) services and MS primarily focus on physical symptoms, only 1 study has investigated how cognitive symptoms and impairments might affect the use of eHealth technologies [12].

### Electronic Health Literacy and Adoption

To achieve successful use, adoption, and value to both participants and researchers in a project with heavy manual data collection, it is necessary to design a useful digital tool with high usability to provide a good user experience to facilitate the adoption [13]. However, the relationship between usefulness and successful use is moderated by participants’ eHealth literacy levels and the system demands on eHealth literacy [13,14]. eHealth literacy can be described as the competencies and skills needed to engage with eHealth tools, and Monkman’s model and Kayser et al’s expanded user-task-context matrix suggest that the design of a digital tool should match the eHealth literacy levels of the target population to ensure successful use and adoption [13,15-17]. The eHealth literacy framework (eHLF) is a multifaceted, conceptual model with 7 distinct dimensions describing eHealth literacy as knowledge, skills, trust, motivation, and user experience with the system aspect. eHLF’s dimensions are (1) ability to process information, (2) engagement in own health, (3) ability to engage actively with digital services, (4) feeling safe and in control, (5) motivation to engage with digital services, (6) having access to systems that work, and (7) digital services that suit individual needs [18].

Although the eHLF contains a dimension that focuses on motivation to use eHealth, a study in symptom management in MS concluded that adoption and completion in an eHealth-based randomized controlled trial (RCT) might have been improved by addressing willingness to participate [19]. This suggests that adoption and successful use in a digital data collection are also influenced by content and purpose. In Deci and Ryan’s self-determination theory, extrinsic motivation involves the individuals’ experience of competence and autonomy as well as relations to others to become motivated [20]. Although the eHLF covers aspects of this, eHealth literacy might be supplemented with additional dimensions focusing on the participants and their relationship with MS, diet, and lifestyle factors.

By complementing the eHLF with selected dimensions from the Health Education Impact Questionnaire (heiQ), we are able to cover aspects of competence and autonomy as well as relatedness. The heiQ is a validated and widely used patient-reported outcomes measure evaluating patient education.

The heiQ consists of 8 dimensions, which describes outcomes related to self-management behavior, and the dimensions have been found to capture aspects strongly related to empowerment [21].

We selected the following 3 dimensions that broaden the motivation aspect from the eHLF: positive and active engagement in life, self-monitoring and insight, and social integration and support [22].

We here report how we have used a combination of the theories of Monkman, eHLF, and heiQ in an analytical framework to identify factors that may be associated with motivation and adoption in a digital data collection relying on manual data reporting from participants with MS.

We use a sequential mixed methods design, which combines interviews, survey, and focus group interviews to gain in-depth knowledge through qualitative phases that are validated in a larger population in a quantitative format.

## Methods

### Study Design

**Figure 1:**
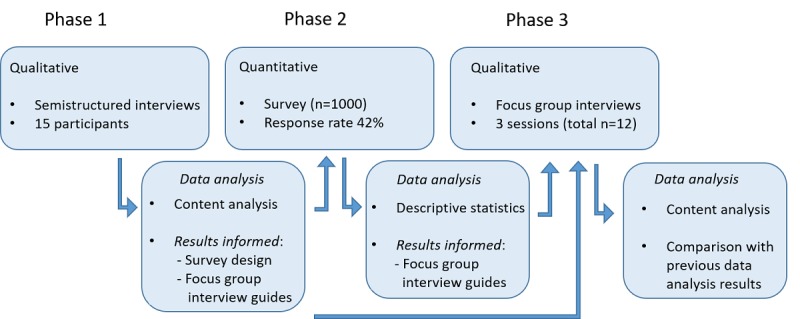
Overview of study design.

In 2016, the Danish MS Patient Society established a research project, the KosMuS project, in collaboration with the University of Copenhagen. The aim was to explore potential correlations between diet and MS disease activity by inviting people with MS to register diet intake, lifestyle factors, and daily changes in MS for up to 100 days. The sequential mixed methods design that was used in this study consists of 3 phases. In phase 1, 15 semistructured interviews were conducted with people with MS. Of 3 categories, 2 identified in the phase 1 analysis were substantiated in a larger population in the phase 2 survey. Findings from phases 1 and 2 described eHealth literacy levels, health behavior, and attitude toward research participation in the participant population. All findings were discussed in 3 focus group interviews in phase 3 to explore how these would and could affect adoption and participation in the KosMuS research project. An overview of the study design is presented in Figure 1.

The study included people diagnosed with MS, Danish speaking, and aged older than 18 years. Severe cognitive impairment and aphasia were exclusion criteria for the interview-based data collections in phases 1 and 3.

### Data Collection

This study’s data collection was conducted together with a second part that explored how individuals with MS experience nutrition to affect their MS disease activity. Following data collection, the 2 parts were handled and analyzed separately. A detailed description of the data collection is presented together with the findings on diet and MS [6].

#### Phase 1: Semistructured Interviews

Guided by the eHealth Literacy Questionnaire and heiQ frameworks, interview guides were based on the 7 dimensions in eHLF and 3 dimensions from the heiQ. The dimensions were chosen by authors AK and LK through discussions that carefully examined the study aim compared with descriptors for each of the dimensions. The selection was furthermore based on other studies’ experiences with using the concepts as frameworks for qualitative studies instead of scale constructs, which both eHLF and heiQ were originally developed for [23].

On the basis of the selected dimensions, authors AK and LK constructed 7 items for the interview guide. Each item and its subtopics for conversation cover 1 to 3 dimensions from eHLF or heiQ. In the interviews, the 7 items were followed by a short introduction to the KosMuS project and 2 items that invited participants to share their thoughts on project design.

A total of 2 pilot interviews were conducted to test the interview guide and the framework’s coverage. Pilot interviews were conducted by author AK, who interviewed 2 individuals with MS at the Danish MS Patient Society’s premises in Valby. Following the interview, the 2 participants provided feedback on the items. Their interview responses and item feedback were combined with interviewer’s notes and then discussed between all 3 authors with consideration to study aim, participants’ experience, framework coverage, and the interviewer’s role. All 9 items were kept in the interview guide, but several item adjustments were made to improve understandability, for example, from “Which *barriers* do you face when using technology?” to “Which *challenges* do you face when using technology in everyday life?” The pilot interviews are not included in the final dataset. A selection of items and their relation to the conceptual dimensions are found in Table 1.

A total of 15 semistructured interviews were conducted from July to August 2016. Participants were recruited through a post on the Danish MS Patient Society’s Facebook page and from the Multiple Sclerosis Hospital in Haslev, Denmark [6]. The interviews were conducted by author AK and lasted 25 to 75 min. All interviews were audio recorded and transcribed by author AK, and all participants were given a pseudonym.

#### Phase 2: Survey

Of 3 categories, 2 identified in the phase 2 content analysis of interviews were further investigated in a survey. The survey format was chosen to investigate these findings in a larger population and a population that was less invested in the study than those who had contacted the research group or accepted an invitation for a face-to-face interview. The survey was distributed with a minimal time gap, and data were gathered to compare data with the findings from the interviews [24].

A draft for survey items was constructed by authors AK and LK based on the findings and overall study aim. The draft was closely scrutinized and adjusted in a meeting with all 3 authors. The authors focused on phrasing the items as closely as possible to the interview findings to ensure alignment [24]. A total of 14 items were constructed for the survey, and an excerpt is presented in Textbox 1. All 14 survey items can be found in Multimedia Appendix 1.

**Table 1:** Examples of interview guide items and their relation to framework dimensions.

Questions	Potential points for conversation	Dimensions
Can you give me 1 or more examples of a technology or app that you like to use and tell a little about what makes it nice to use?	Design, functionality, aids, difficulty level/skills	eHLQ^a^—dimension 3: ability to actively engage with digital services eHLQ—dimension 7: digital services suit individual needs
What kind of support do you have in everyday life? Related or unrelated to MS^b^.	Family and friends, assistants and health professionals, technology, tools and aids	heiQ^c^—dimension 1: the positive and active engagement in life heiQ—dimension 7: social integration and support

^a^eHLQ: eHealth Literacy Questionnaire.

^b^MS: multiple sclerosis.

^c^heiQ: Health Education Impact Questionnaire.

**Excerpt of survey items sent out to 1000 individuals with multiple sclerosis.**

In everyday life, I need to ration my energy because of multiple sclerosis (MS)

- Strongly disagree
- Disagree
- Agree
- Strongly agree

What would be the most important reason(s) for participating in a research project like this? Choose up to 3 of the following options.

- To contribute to research in MS
- To learn more about myself and my MS
- To assist in weight loss
- To pass on my own experiences about MS and diet
- To exchange experiences with other people with MS
- To be part of the research project (planning, etc.)
- To learn more about nutrition and lifestyle

The survey was constructed and managed digitally via SurveyXact [25]. It was sent out by email to a randomized sample of 1000 people from the Danish MS Society member database [6]. A reminder was sent to nonrespondents after 10 days.

#### Phase 3: Focus Group Interviews

The final phase of this study consisted of 3 focus group interviews that explored how findings from phases 1 and 2 could inform project design and development of the digital data collection tool.

The group dynamics in focus group interviews allows for an often deeper and richer data material, created through the social interaction facilitated by the interviewer [26]. All 3 interviews included an exercise, in which participants were asked to collaboratively rank and discuss reasons and incentives for participating in a digital health research project on nutrition and MS. The project would contain daily registrations on symptoms, diet intake, and lifestyle factors, which would have to be registered manually through a smartphone-based digital data collection tool.

The 3 focus group interviews were conducted in the Danish MS Society in Valby, Denmark (3, 4, and 5 participants in each) in January 2017 [6]. Participants were recruited from a list of people who had signed up for receiving updates on the KosMuS study. Interviews lasted 90 to 125 min and were recorded on Dictaphone and later transcribed by author AK [6].

### Data Analysis

#### Semistructured Interviews

Data were analyzed using content analysis [27]. This method allowed for a deductive coding of statements and topics related to eHealth literacy, empowerment, or health behavior dimensions while still leaving room for the inclusion of inductively identified categories. Interviews were coded in NVivo by author AK [28]. After coding of the first 3 interviews, the coding strategy was carefully reviewed and discussed with author LK. These 3 interviews were then recoded based on the adjusted coding strategy. Codes were printed out on paper and grouped into categories that were named. This work was conducted by author AK, followed by 2 sessions in which all 3 authors participated and reviewed codes and categories.

#### Survey

Basic descriptive statistics were used to identify response patterns [29]. Summarized responses were then compared with the earlier findings from the interviews.

#### Focus Group Interviews

The transcribed focus group interviews were deductively coded according to identified categories from the analysis of semistructured interviews. Coding was conducted in NVivo [28]. Coding strategy and excerpts of coded material were discussed with authors LK and LS. Code content was compared with the findings from previous phases, and the results were discussed between the authors. On the basis of this, the results were divided into factors that could encourage or discourage participation.

### Ethical Considerations and Data Agency

In this study, no biological material or medical devices were used, and the participants were not subjected to any kind of diagnostics or treatment. Consequently, approval from the Danish National Committee on Health Research Ethics (Den Nationale Videnskabsetiske Komite) was not required, which is the case for all studies only involving interviews and questionnaires [30].

The study was registered and approved with the Danish Data Protection Agency (2016-41-4723).

## Results

### Participants

A total of 5 men and 10 women, with a mean age of approximately 47 years (ranging from 28 to 69 years), accepted an invitation to an individual interview in phase 1. In the phase 3 focus group interviews, 2 men and 10 women with a mean age of approximately 50 years (range: 26-63 years) participated. Overall, 78.5% (334/425) of the survey participants were women, and the mean age among all survey participants was approximately 52 years (range: 19-77 years). The participants across all 3 phases represented the varying types and stages of MS, and they had, on average, been diagnosed for approximately 12 years. Each interview participant only participated in 1 interview activity, whereas the randomized sample used for the survey did not consider earlier participation. An overview of participants’ sociodemographic distribution is shown in Table 2.

Overall, 7 of the interview participants in phase 1 and all 12 participants in phase 3 were invited from a list of individuals with MS who had signed up for updates on the KosMuS project on nutrition and MS. In addition, 8 of the interview participants and all survey participants were invited without having shown prior interest in KosMuS. Categories identified in the interview analysis (phase 1) were present among both those who signed up by themselves and those who participated after being invited. However, participants who had indicated interest in the project and considered themselves likely participants in the KosMuS project were less concerned of overcoming factors that might act as barriers.

### Findings From Phase 1 Interviews and Phase 2 Survey

The content analysis of the phase 1 interviews identified the following 3 categories: (1) *life with MS*, (2) *use of technology, and* (3) *participation and incentives.* The first category represents the context of living with MS and how it affects everyday life. Category 2 relates to the everyday use of technology with regards to both skill and attitude toward the use. The final category contains participants’ thoughts on and motivations for participation in research projects in general and the KosMuS project specifically.

In the following, each category is presented together with the findings from the survey in phase 2.

These are followed by a presentation of the findings from phase 3 focus group interviews, which are summarized focusing on factors that encourage or discourage adoption and participation.

An overview of categories identified in the content analysis can be found in Table 3, and survey results are summarized in Table 4.

#### Life With Multiple Sclerosis

The analysis of the semistructured interviews showed that all participants were affected by their MS in everyday life. Factors related to life with MS that were identified as potential influencers on the participation in a research project with digital data collections were divided into the following 3 subcategories: (1) physical and cognitive limitations, (2) disease management in everyday life, and (3) the social aspect and sharing with other. Table 3 provides an overview of each category. In the survey, 73.4% (n=245) participants answered that they experienced fatigue on a daily basis, and 78.2% (n=332) related daily changes in energy levels to MS. When responding to the statement “I feel limited because of my MS,” 74.2% (n=315) answered agree or strongly agree, 84.3% (n=358) of the respondents said that they felt the need to ration their energy on days where they experienced MS symptoms, and 46.4% (n=197) said that they sometimes turn down arrangement or social events because of the MS. Table 4 provides an overview of survey results.

Although the analysis of the interviews indicated that participants found the disease-modifying drugs to be a necessary evil, less than a fourth (23.5%, n=100)) of the survey respondents agreed that taking medication affected their daily quality of life.

Survey responses supported the interview analyses with 89.0% (n=377) not only agreeing that social support was important to them in relation to their MS but also considered it their own responsibility to learn how to live with their MS (97.4% agree or strongly agree, n=416). The ambivalence of interacting with other people with MS was reflected in response to the statement “It means a lot to me to participate in networks (with other people with MS),” with 39.5% (n=168) responding agree or strongly agree, and 60.3% (n=257) disagree or strongly disagree.

**Table 2:** Sociodemographic distribution of participants in interviews, survey, and focus group interviews.

Characteristics		Phase 1 (N=15), n	Phase 2 (N=425), n (%)	Phase 3 (N=12), n
**Sex**				
	Female	10	334 (78.5)	10
	Male	5	91 (21.4)	2
**Age (years)**				
	18-29	1	14 (3.3)	1
	30-39	3	64 (15.1)	1
	40-49	5	100 (23.5)	2
	50-59	5	135 (31.8)	5
	60-69	1	87 (20.5)	3
	70-79	—^a^	25 (5.9)	—
**Type of MS^b^**				
	Relapse remitting	9	257 (60.5)	8
	Secondary progressive	2	57 (13.4)	2
	Primary progressive	3	74 (17.4)	2
	Do not know	1	37 (8.7)	—
**Year of diagnosis**				
	1989 or earlier	—	41 (9.6)	—
	1990-1999	2	84 (19.8)	3
	2000-2009	7	158 (37.2)	8
	2010 or later	6	142 (33.4)	1

^a^Not applicable.

^b^MS: multiple sclerosis.

**Table 3:** An overview of categories and subcategories from phase 1 semistructured interviews.

Category and subcategory		Content
**Life with MS^a^**		
	Physical and cognitive limitations	For some participants with more severe disease progression, physical and cognitive symptoms were part of everyday life, whereas other participants primarily experienced severe symptoms during relapses or stressful periods. Physical symptoms included, but were not limited to, decreased walking ability, fine motor skills impairment, visual impairment, and digestive issues. A majority of participants experienced a lack of energy and fatigue and had to ration their resources and avoid unnecessary stress: “[I] try to say no to things that I would have liked to participate in. But where I know that right now my system needs rest.” [Female, 31 years, diagnosed in 2014, ID: 1.5] “If I’m expected to do something. At a certain time, and I’m running late. Then I become desperate. Because... They [legs] just go numb.” [Female, 51 years, diagnosed in 2012, ID: 1.1]
	Disease management in everyday life	The majority of participants with relapse-remitting MS were in disease-modifying treatments. However, participants on disease-modifying drugs often experienced harsh side effects: “Because my experience is that the medication has so many side effect that the quality of life is more affected by the medication than by the MS.” [Female, 63 years, diagnosed in 2001, ID: 1.4] “You’re completely trapped in ‘Should I stay or should I go’. All the time. Because you know that the chemistry in that medication is awful, but on the other hand, you have no idea what happens and a lot happens with this disease all the time, and you’re constantly reminded of it.” [Male, 51 years, diagnosed since 2012, ID: 1.6] Both participants in and without disease-modifying treatments used complementary treatments and lifestyle to relieve symptoms or disease activity and increase emotional well-being. Participants underlined that they considered it their own responsibility to have a good life and cope with the disease. This point of view was mainly expressed by participants who had made active decisions on lifestyle and complementary treatments following the MS diagnosis.
	The social aspect and sharing with others	The majority of participants (12 of 15) had social media accounts and used services such as Facebook and Instagram daily. These accounts were used to stay in contact with family and friends and participate in digital MS patient networks. To other participants, networks and groups on especially social media negatively increased their awareness of the disease. Participants with few MS symptoms found that the groups were too focused on disease, and on the contrary, participants who had been diagnosed for more years found it discouraging when other people with MS had higher functional levels than themselves: “For example. There’s one [Facebook group] that is about exercise and MS. [...] But among the members was someone who was competing in Miss Fitness or something. She worked out constantly. And hard workouts. She worked out like I used to do. And it was just depressing for me. And I felt like that kind of posts weren’t really appropriate for an MS page.” [Female, 43 years, diagnosed in 2014, ID: 1.2]
**Use of technology**		
	Widespread use	All participants used computers and cellphones in everyday life. Of 15 participants, 14 owned and used a smartphone, and most participants had access to both computers and tablets. Smartphone-based technology was considered not only positive for its ability to facilitate easy communication with social network but also negative because of the constant interruptions and the expectation of constantly being online: “But I can sometimes dream about taking my smartphone and conducting a small memorial service for it and say thank you. And then throw a rose on top. But you don’t do something like that, I know. Because all the kids [grandchildren] go calling me on it.” [Male, 70 years, diagnosed since 2004, ID: 1.11] “You just have to see if there’s something, and to see if you’re important. You’re not. I think it has become too much.” [Female, 51 years, diagnosed in 2012, ID: 1.1]
	Preferred design and usability	When participants described apps or Web-based apps they enjoyed using, keywords included the following: simple design, accessibility (preferably with 1-point entry to all needed functions), easy overview, usefulness
	Barriers	While some participants related these preferences to their digital skill level or personal taste, others found it necessary because of their MS. One participant used the term *screen clutter* to describe digital services that she felt were hard to use. Several participants, mostly among those who had been diagnosed for more years, described problems with small fonts, many colors and ads, and small buttons. One younger participant underlined that despite barriers, she was not interested in aids or special solutions: “The problem is not that there aren’t phones with bigger screens. It’s just because I don’t want to look disabled.” [Female, 41 years, diagnosed in 1997, ID: 1.10]
	Technology as an aid	Participants especially used reminder apps to remember medication, grocery lists, calendar appointments, and general reminders. Two participants used memory game apps with the purpose to prevent cognitive decline. One participant used a spreadsheet to keep track of side effects and disease progression.
**Participation and incentives**		
	Motivation for participation	The main motivation for participation in a digital data collection was to contribute to research. Other motivations included a personal interest in nutrition and lifestyle, weight loss, contributing with own knowledge and experiences to get a more positive perspective on MS, contributing with own experiences and learn more from other people’s experiences, to gain knowledge about yourself, and to find out something useful in cooperation with others: “Because I do research in my disease everyday and learn something new from living with it. [...] And I would like to share my knowledge.” [Female, 63 years, diagnosed in 2001, ID: 1.4] “The biggest motivation would actually be that Now we really managed to make something really good that others can benefit from, and that I have participated in that.” [Female, 41 years, diagnosed in 2002, ID: 1.8] “I think it [diet and nutrition] works for me. And I have no doubts that I have to participate in something like this” [Male, 51 years, diagnosed since 2012, ID: 1.6]
	Expectations to participation	Of 15 participants, 12 stated that they would be interested in participating. Participants who considered nutrition and lifestyle to affect MS were more likely to express the intention to participate in the research project. Although the majority would like to participate, participants’ main concern was related to the complexity and daily time consumption of diet registrations: “I’m wondering if it will be too much of a hassle, and if you’ll get it done [the daily registration].” [Female, 39 years, diagnosed in 2009, ID: 1.12]

^a^MS: multiple sclerosis.

**Table 4:** Overview of survey results (N=425).

Indicate how much you disagree or agree with the following statements	Strongly disagree, n (%)	Disagree, n (%)	Agree, n (%)	Strongly agree, n (%)
When I was diagnosed, there was a time where it was difficult to relate to anything else than the disease	38 (8.7)	95 (22.4)	146 (34.4)	146 (34.4)
I feel limited because of my MS^a^	35 (8.2)	75 (17.6)	202 (47.6)	113 (26.6)
I need to ration my energy in everyday life because of my MS	35 (8.2)	32 (7.6)	175 (41.1)	183 (43.2)
I often say no to things because of my MS	78 (18.4)	150 (35.3)	132 (31.1)	65 (15.3)
I use reminders and/or calendar to remember appointments and tasks	38 (8.7)	49 (11.8)	169 (39.7)	169 (39.7)
I am often in doubt if my symptoms are caused by MS	39 (9.2)	134 (31.6)	170 (40.0)	82 (19.2)
If I take MS disease-modifying medication, my quality of life decreases	173 (40.8)	152 (35.5)	65 (15.3)	35 (8.2)
I make an effort to avoid that my MS makes me appear different from others	27 (6.3)	75 (17.6)	193 (45.5)	130 (30.3)
It is important to me that I experience social support, when I need it	6 (1.3)	41 (9.5)	217 (51.1)	161 (37.9)
It means a lot to me to participate in networks (with other people with MS)	69 (16.1)	188 (44.2)	127 (30.0)	41 (9.5)
It is my responsibility to learn to live with MS	3 (0.7)	6 (1.3)	176 (41.3)	240 (56.3)

^a^MS: multiple sclerosis.

#### Use of Technology

The analysis showed that participants all used technologies such as smartphones or computers in everyday life. Technologies were used to communicate with family and friends, as an aid (reminder and calendars), and to share experiences with others with MS. Although most participants had positive attitudes toward technologies, a few found it to be antisocial and frustrating to have to use.

In the survey, 79.4% (n=338) said that they used (digital) tools (eg, calendars and reminders) to help them in everyday life. These responses were in line with the findings from the interview analyses. Moreover, 75.8% (n=323) agreed to the statement “I make an effort to avoid that MS makes me appear different from others.”

#### Participation and Incentives

Participants’ reflections were mainly divided into thoughts on participation and motivations for participating. Primary motivations for participation included *to contribute to research, an interest in the topic (here diet and nutrition),* and *to contribute and share own knowledge.* Although participants were motivated to participate in a large-scale digital data collection, they expressed worries about the extensiveness of the registrations and the time consumptions. An overview of the results is found in Table 3.

In the following survey, when asked about if respondents could imagine participating in a project such as KosMuS, 20.7% (n=88) answered definitely; 8.8% (n=37) yes, depending on how good the app is; 27.4% (n=117) yes, depending on the workload; and 43.0% (n=183) answered that they would not be interested in participating.

Of those who were interested in participation, the main motivations for participation were listed as the contribution to research and the possibility of learning more about themselves and their MS. When asked to estimate an acceptable workload per day, participants’ answer ranged from 4 to 60 min, with an average of 16 min.

### Focus Group Interviews: Finding the Motivation

In each focus group interview, the KosMuS project was presented together with the findings from phases 1 and 2. Participants then discussed how the findings in each of the 3 categories could encourage or discourage participation in the project and adoption of the tool for digital data collection.

All 12 focus group interview participants considered themselves potential participants. However, the majority of participants emphasized that there were a number of conditions that would have to be met for them before they would enroll.

In the following sections, findings from the focus group interviews are grouped into reasons for not participating and motivations to participate.

#### Reasons for Not Participating

Some participants stated that they had plenty of time to spare in daily life, but for the majority of participants, the amount of time spent daily on registrations would be a crucial factor for their decision to enroll in the research project. Available time that could be allocated was affected by family, work, and parts related to living a life with MS symptoms. All participants agreed with the survey response that indicated a maximum of 15 min per day.

The results of the analysis indicated that when talking about the importance of daily time consumption in the project, participants wanted simple registrations that were convenient and not considered invasive in relation to their everyday life routines. Participants particularly emphasized the importance of convenience in a project such as KosMuS that stretches over several months. This also included being able to do everything from home:

The biggest obstacle to me is if it’s one of those research project where you have to have blood samples taken and show up for thing all the time.

Female, 44 years, diagnosed in 2005, ID: 3.1

I agree. [...] I would think that it was a problem to take time off from work. Spend the whole day on it.

Female, 37 years, diagnosed in 2008, ID 3.5

Convenience was also a main priority for participants when talking about potential registration modes:

I don’t use my computer every day, but I do use my smartphone.

Male, 51 years, diagnosed since 2012, ID: 3.6

Participants favored smartphones because of the flexibility that allowed participants to register on the go and not worrying about bringing papers or computers with you.

Participants were not scared off by daily registrations, but they did express worries about the complexity and detail of registrations:

The thing about... That now I’m gonna have yoghurt. Then I go into the app and choose yoghurt, but then I have to weigh my yoghurt, and then I have to find the scale. I don’t know.

Female, 51 years, diagnosed since 1995, ID: 3.8

Participants were interested in easy registrations where it was okay to make estimates of portion size. In the third interview, they also emphasized the importance of this in relation to *bad days with MS*. Days where symptoms increased might be not only the most important ones to register but also the hardest for the participants to find the energy to do so.

Talking about nutrition and the hypotheses on MS being affected by nutrition, several participants raised the concern that projects such as KosMuS might attract people who are already interested in MS diets, and that people with normal eating patterns would choose not to participate because they did not feel that they were eating right:

There might be some that live such unhealthy lifestyles that they don’t want anyone to see or get involved. [...]

Female, 37 years, diagnosed in 2008, ID 3.5

Their inputs could be really important too.

Male, 61 years, diagnosed in 2008, ID: 3.4

That’s the thing.

Male, 61 years, diagnosed in 2008, ID: 3.3

It’s all about telling them that it’s okay for them to live the way they do. And that they can still contribute with valuable information.

Male, 61 years, diagnosed in 2008, ID: 3.4

Participants emphasized that it would be important to provide clear information on what it takes to participate in the project and that communication before, during, and after participation would be crucial. Before the project communication should contain information on how to get started and during the project period, several participant discussions focused on the importance of knowing that someone was receiving the information they registered, and that it was a valuable contribution toward the project aim:

Support and feedback when we’re registering. Quietly from the side line.

Male, 51 years, diagnosed since 2012, ID: 3.6

To know that all we register is going to be used.

Female, 54 years, diagnosed in 2006, ID: 3.9

Yes, and that you have received it, so it has not just flown out into the blue.

Female, 51 years, diagnosed since 1995, ID: 3.8

That would give some motivation and energy, it would.

Female, 54 years, diagnosed in 2006, ID: 3.9

#### Motivation to Participate

A total of 7 participation incentives that had been identified in the interviews were handed out to each focus group and were in collaboration listed by importance to the participants (see results summary in Textbox 2).

When asked to rank participation incentives, the following 3 incentives were the highest ranked across the focus group interviews: *to contribute to research*, *to learn more about myself and my MS*, and *to exchange experiences with other people with MS*.

When describing the third-highest ranked incentive about exchanging information with other participants with MS, several individuals talked about good experiences, and that they considered it healthy in general to talk to others in the same situation. For others, the exchange of experiences was a way to learn about things that had helped others:

I would still argue for this one. That you exchange information. Because that would give me some insight. [...]

Male, 31 years, diagnosed since 2008, ID: 3.3

And that’s also a thing which can help others. Because I think all your inputs [gestures toward other participants] are really interesting, and then I can go home and read up on it.

Female, 37 years, diagnosed since 2008, ID: 3.5

Focus group 2 inserted an additional category *Feedback* that they described as getting feedback during the project and after the project had been concluded.

Participants, in general, agreed that weight loss would not be a personal motivation for participating in the research project, and *to assist in weight loss* was prioritized last among motivations in all 3 groups.

**Collectively ranked motivations for participation. MS: multiple sclerosis; a: Category added by the focus group interview participants.**

Focus group interview 1

- To contribute to research in MS
- To learn more about myself and my MS
- To exchange experiences with other people with MS
- To learn more about nutrition and lifestyle
- To pass on my own experiences about MS and diet
- To be part of the research project (planning)
- To assist in weight loss

Focus group interview 2

- To contribute to research in MS
- *To receive feedback during and after participation^a^*
- To exchange experiences with other people with MS
- To learn more about myself and my MS
- To learn more about nutrition and lifestyle
- To pass on my own experiences about MS and diet
- To be part of the research project (planning)
- To assist in weight loss
- To be part of the research project (planning, etc.)

Focus group interview 3

- To learn more about myself and my MS
- To contribute to research in MS; to be part of the research project (planning)
- To exchange experiences with other people with MS
- To learn more about nutrition and lifestyle
- To pass on my own experiences about MS and diet
- To assist in weight loss

## Discussion

### Principal Findings

Our findings show that when organizing and designing a tool for digital data collection in research projects for people with MS, there are disease-specific implications that are likely to affect the adoption and accessibility. Cognitive and physical symptoms related to MS such as vision impairment, tremors, and dizziness/fatigue might lower the accessibility if the digital tool is not suited to fit the needs caused by various MS symptoms. Despite limitations caused by MS symptoms, our findings indicate a high level of technology use in the Danish MS population, and participants in this study used smartphones for both everyday life communication and as MS aids—for example, reminders and alarms.

The adoption of a digital tool together with the research project itself is not affected by the disease itself. Family, friends, and peers with MS affect how individuals with MS use technologies and, particularly, smartphone-based solutions in everyday life. Medication and its side effects together with the uncertainty of the disease affect the willingness to participate in a project such as KosMuS.

Worries and reasons not to participate are primarily linked to the content and workload of the project and not so much the digital tool itself that provides the convenience and flexibility of not having to show up on particular times and places.

Our results indicate a positive attitude among people with MS toward participating in research. The primary incentive was the contribution to research and in the long run to contribute to new knowledge on how to better manage MS. This was closely followed by the wish to learn more about oneself and what affects one’s own MS and to share experiences and advice with other people with MS.

For people with MS to enroll in a quite extensive research project such as KosMuS, the tool needs to be convenient and easy to use. It should be stated clearly what is expected of the participants, and our findings indicate that the communication between project coordinators and participants and the feedback to participants are equally important to participants compared with the design of the digital tool itself.

#### Previous Research

Few studies have investigated the implications of MS when designing eHealth or other technologies for an MS population. Atreja et al published a qualitative study in 2005 that informed on the design of a Web portal for individuals with MS [12]. The authors identified similar barriers to using a digital tool because of physical limitations caused by MS. However, the study was conducted before the introduction of smartphone technologies and large-scale social media. Although some of the limitations remain the same, Web 2.0 has changed accessibility, for example, smaller screens affect people with vision impairment, and small buttons on a touch screen affect people with fine motor skills impairment.

Although we have not been able to identify other studies investigating the motivation for participation in research among people with MS, Carroll et al explored motivations for participation in RCTs among people with pulmonary arterial hypertension [31]. Similarly, their results showed that major motivations were related to both personal interests (eg, getting better) and altruistic motives (eg, contributing to research). However, compared with our study, the motivational factors identified by Carroll et al are more focused on clinical aspects (eg, safety) than the learning experience (eg, learning more about one’s own MS). This might be because of different research contexts (RCT vs observational digital data collection) or different diseases.

This study used dimensions from eHLF and the heiQ to investigate factors that affect the adoption and actual use of the eHealth tool as well as participants’ willingness to participate in a project such as KosMuS. The dimensions covered in the interview guide are to an extent represented in the findings. Dimensions such as *ability to actively engage with digital services (eHLF, dimension 3), digital services that suit individual needs (eHLF, dimension 7), engagement in own health (eHLF, dimension 2), and self-monitoring and insight (dimension 5, heiQ),* are clearly reflected in the identified categories’ content. On the contrary, *feel safe and in control (eHLF, dimension 4)* is less evident in the findings. The dimension was included in all interviews, but the topic did not spark any elaboration or clear opinion on data safety and trust among the participants. This might be because the dimension is not a matter of concern to the participants. The finding is in line with a study on eHealth literacy in a Danish outpatient clinic population that also observed less concern about eHLF’s dimension 4 [32].

#### Methodological Considerations

Participants in all 3 phases of the study signed up voluntarily, and all focus group interview participants and half of the individual interview participants had already indicated an interest in the KosMuS project before being invited to interviews. This might indicate that parts of our study population have an existing interest in nutrition, research, and eHealth. Identified motivational factors in this study such as *to exchange experiences with others with MS* and *to learn more about myself and my MS* might not be applicable to potential participants who do not have an interest in nutrition or self-management. Therefore, the data collection participants might also be more interested in nutrition and self-management, which may affect the collected data, for example, with under-/overrepresentation of different nutrition patterns.

A majority of participants in all 3 phases were female. This is consistent with the background MS population, in which more than 2 of 3 participants who diagnosed with MS are female [33,34].

Using a sequential mixed methods design, we have been able to continuously qualify and strengthen our results. However, we acknowledge that the researchers’ subjectivity is an integrated part of the research process [35]. We have encouraged discussions of this matter in our frequent meetings and in both the processes of design and analysis. Using a framework for the interview, we risk guiding the participants too much, but the framework has also made it easier to address the introduction of elements into the interview guides, survey design, or analyses that were not in line with the original study aim.

There might also be underlying factors that those interested in nutrition and lifestyle are also the ones that are more affected by the side effects from medication. Although medication side effects were a big part of the interviews, only 23.5% (n=100) agreed to the statement that medication affects the quality of life negatively.

#### Recommendations

Our findings support the hypothesis that a system’s design should be adjusted to meet the eHealth literacy levels of the user group [13]. In our study, we have used an analytical framework with a combination of dimensions from the eHLF and the heiQ. Future research should further explore how the combination of eHealth literacy and empowerment-related dimensions might assist the development and implementation of digital tools focused on disease management and other patient groups.

It is important to acknowledge that in projects similar to the one described here, the participant burden is high; that is, the project organizers need to contribute with extra work to avoid imbalance. The results from this study should be incorporated into the development of the eHealth tool for the KosMuS project. However, several of these findings are disease specific and not related to this particular project. When designing eHealth-based solutions for people with MS, factors related to the disease and living with the disease should be included in the design phase.

### Conclusions

The interviews and survey in this study identified 3 categories that are important to address in the design of an eHealth-based research project in a population of individuals diagnosed with MS: life with MS, use of technology, and participation and incentives. The focus group interviews furthermore identified *to contribute to research, to learn more about one’s own MS, and to share experiences with others* as main motivational factors for participation. These factors should be taken into consideration in the design of a study dependent on user-generated data in an MS population and in the development of a digital tool for data collection.

## Appendix

Multimedia Appendix 1 Survey items.

## Abbreviations

eHealth: electronic health
eHLF: eHealth literacy framework
heiQ: Health Education Impact Questionnaire
MS: multiple sclerosis
RCT: randomized controlled trial
