# Supplementary material for: Identification of Factors That Motivate People With Multiple Sclerosis to Participate in Digital Data Collection in Research: Sequential Mixed Methods Study
Source: JMIR Hum Factors. 2019 Oct 9;6(4):e13295. doi: 10.2196/13295 (PMC6812479; doi:10.2196/13295)
Supplement: Multimedia Appendix 1 [file humanfactors_v6i4e13295_app1.pdf]

## Appendix 1 – Survey items

*The original survey was conducted in Danish, and the items below are non-validated translations.*

# Life with MS

In the following questions, please respond based on your own experience with living with MS.

| **How strongly do you disagree or agree with the following statements:** | **Strongly disagree** | **Disagree** | **Agree** | **Strongly agree** |
|---|---|---|---|---|
| When I was diagnosed, there was a time where it was difficult to relate to anything else than the disease | ❑ | ❑ | ❑ | ❑ |
| I feel limited because of my MS. | ❑ | ❑ | ❑ | ❑ |
| I need to ration my energy in everyday life because of my MS | ❑ | ❑ | ❑ | ❑ |
| I often say no to things because of my MS | ❑ | ❑ | ❑ | ❑ |
| I use reminders and/or calendar to remember appointments and tasks | ❑ | ❑ | ❑ | ❑ |
| I am often in doubt if my symptoms are caused by MS | ❑ | ❑ | ❑ | ❑ |
| If I take MS disease-modifying medication, my quality of life decreases | ❑ | ❑ | ❑ | ❑ |
| I make an effort to avoid that my MS makes me appear different from others. | ❑ | ❑ | ❑ | ❑ |
| It is important to me that I experience social support, when I need it | ❑ | ❑ | ❑ | ❑ |
| It means a lot to me to participate in networks (with other people with MS) | ❑ | ❑ | ❑ | ❑ |
| It is my responsibility to learn to live with MS | ❑ | ❑ | ❑ | ❑ |

In the KosMuS study, we aim to investigate the potential correlations between MS and lifestyle factors. The study is based on data collected from people with MS and it includes data on diet, MS symptoms, physical activity, stress and sleep. Participants are asked to make daily reports on these factors.

Responses to the questions below are used to design the data collection, and will not be considered as a formal sign up to the study.

# Data collection in the KosMuS study

1. How many minutes a day would it be possible for you to spend on daily registrations for a research project like KosMuS?

Approximately________ minutes

2. What would be the most important reasons for participating? Choose up to three of the options below.

- ❑ To contribute to research in MS
- ❑ To learn more about myself and my MS
- ❑ To assist in weight loss
- ❑ To exchange experiences with other people with MS
- ❑ To pass on my own experiences about MS and diet
- ❑ To be part of the research project (planning, etc.)
- ❑ To learn more about nutrition and lifestyle

3. Could you imagine that this would be a project that you would like to participate in?
   - ❑ Yes, definitely
   - ❑ Maybe, depending on the workload
   - ❑ Maybe, depending on the digital tool that is used to report data in
   - ❑ No

**Questions and comments**

______________________________________________________________________________

______________________________________________________________________________

______________________________________________________________________________

____________________________________________________________
